# The Prevalence and Risk Factors of Hypertension among the Urban Population in Southeast Asian Countries: A Systematic Review and Meta-Analysis

**DOI:** 10.1155/2021/6657003

**Published:** 2021-02-10

**Authors:** Azmawati Mohammed Nawi, Zulkefley Mohammad, Kavita Jetly, Mohamad Aznuddin Abd Razak, Nur Suhada Ramli, Wan Abdul Hannan Wan Ibadullah, Norfazilah Ahmad

**Affiliations:** ^1^Department of Community Health, Faculty of Medicine, Universiti Kebangsaan Malaysia, Jalan Yaakob Latif, 56000 Cheras, Kuala Lumpur, Malaysia; ^2^Health Services Division, Malaysian Armed Forces Headquarters, JalanTekpi, Off Jalan Padang Tembak, 50634 Kuala Lumpur, Malaysia; ^3^Ministry of Health (Malaysia), Federal Government Administrative Centre, 62514 Putrajaya, Malaysia

## Abstract

The trend of global prevalence for hypertension has been dramatically increasing for the past two decades in Southeast Asian countries. A systematic review aiming to assess the prevalence of hypertension and its risk factors among the urban population in Southeast Asian countries was conducted. We performed database searches of PubMed and Web of Science and performed meta-analysis to determine the pooled prevalence estimate. The overall pooled prevalence estimate of hypertension for Southeast Asian urban population was 33.82%. Among this, 33.98% of hypertension was reported in the community and 32.45% among adolescents in school. The common risk factors that we found were male, ethnicity, education and socioeconomic level, body mass index, waist circumference, smoking, and dyslipidaemia. The review indicates an urgent need for primary and secondary prevention activities. Therefore, a multisectoral and intersectoral approach and collaboration should be undertaken to improve the overall health outcomes of all populations in all Southeast Asian countries.

## 1. Introduction

Hypertension is a significant public health concern and is one of the major causes of premature death worldwide. An estimated 1.13 billion people worldwide have hypertension. In 2015, a survey showed that 1 in 4 women and 1 in 5 men have hypertension. Fewer than 1 in 5 people have well-controlled hypertension [1], and more than 9 million deaths are associated with hypertension [2].

Hypertension is diagnosed if, when it is measured on two different days, the systolic blood pressure reading on both days is ≥140 mmHg and/or the diastolic blood pressure reading on both days is ≥90 mmHg. Well-managed and well-controlled hypertension leads to better quality of life and reduces the risk of complications, which include coronary artery disease, heart failure, cerebrovascular disease, and chronic kidney disease [3].

Based on the recent publication on the update of pediatric clinical practice guidelines (CPG) by the American Academy of Pediatrics, the definition of hypertension in adolescents of age more than 13 years of age is a systolic of ≥130 mmHg and/or a diastolic of ≥80 mmHg [4]. The blood pressure measurement is needed to be taken on separate clinic visits to confirm the diagnosis of hypertension among adolescents. Early detection, early diagnosis, and maintaining healthy lifestyle including promoting physical activities, no smoking, and accurate medication have been shown to help prevent and control hypertension among adolescents [5].

The trend of global prevalence for hypertension has been dramatically increasing for the past two decades. Globally, at least 1 billion people have hypertension, and a projected figure of 1.5 billion is expected by 2025 [2]. Southeast Asia (SEA) is a subregion in Asia. The SEA countries are Malaysia, Indonesia, Thailand, Singapore, the Philippines, Vietnam, Laos, Cambodia, Myanmar, and East Timor [6]. The global epidemic of hypertension has not spared the SEA countries. About one-third of SEA adults have currently been diagnosed with hypertension, and an estimated 1.5 million deaths are associated with hypertension annually [7].

The risk factors for developing hypertension are divided into modifiable and nonmodifiable risk factors. The modifiable risk factors include diet, physical activity, alcohol consumption and tobacco smoking, and obesity or overweight. In contrast, the nonmodifiable risk factors include family history of hypertension, age >65 years, and the presence of other comorbidities, including diabetes and chronic kidney diseases [8].

The relationship between hypertension and socioeconomic status has been extensively studied worldwide. For example, a study conducted in China showed that the prevalence of hypertension is 80.6% (urban, 76.6%; rural, 82.2%), but the authors found that the increment rate was higher (27.9%) in urban areas than in rural areas (25.7%) [9]. As the world is currently experiencing rapid technological advancements and the urban population is increasing, people living in urban areas may experience epidemiological changes, especially with regard to health issues, hence bringing noncommunicable diseases such as hypertension and other cardiovascular diseases into the limelight. The matrix process of urbanisation has been apparent in changing lifestyles towards becoming more sedentary, and this is worsened by an unhealthy diet, smoking, and alcohol consumption. These are the significant risk factors that could contribute to the development of hypertension among the urban population [10].

Focusing on the SEA countries, which are currently experiencing rapid modernisation and fast-changing lifestyles and approaching the developed and urbanised countries, their populations might exhibit epidemiological changes in terms of trends and risk factors of developing hypertension. To our knowledge, limited studies have evaluated the prevalence of hypertension in SEA urban areas. Therefore, we conducted this systematic review and meta-analysis to bridge the knowledge gap mainly related on the current hypertension prevalence and the main contributing factor to it. The objective of our systematic review is to determine the prevalence of hypertension and to identify its risk factors among the urban population in SEA countries.

## 2. Materials and Methods

### 2.1. Bibliography Search Strategy

We conducted this systematic review according to the recommendations of the Preferred Reporting Items for Systematic Reviews and Meta-Analyses (PRISMA) guidelines published by Moher et al. [11]. Using Boolean phrases, we identified studies that reported the prevalence of hypertension among the urban population in SEA countries. We searched and identified published studies using automated database searches of PubMed and Web of Science (WoS). WoS is a robust database consisting a multidisciplinary category including medical and public health. PubMed is the second database used in this review due to huge compilation medical-related articles. The lists of retrieved references were articles published between January 2015 and April 2020. For the PubMed searches, we used medical subject headings (MeSH) terms and advanced search builder features. We used the following terms and variations on these keywords: (“prevalence” OR “incidence”) AND (“risk factors” OR “factor” OR “factors”) AND (“hypertension” OR “high blood pressure” OR “cardiovascular disease” OR “CVD” OR “myocardial infarction” OR “ischemic heart disease” OR “coronary artery disease” OR “cardiac arrhythmia” OR “cardiomyopathy”) AND (“urban” OR “town” OR “city”).

### 2.2. Study Inclusion and Exclusion Criteria

First, we screened studies through a title brief review to check their relevancy and removed duplicates. Five authors performed the title screening, followed by a detailed abstract screening and full-text review to determine the outcome of interest and other inclusion requirements. The study inclusion criteria were as follows: (i) cross-sectional, cohort, or survey studies only. (ii) Sample size and number or prevalence of hypertension was clearly stated. (iii) Full-text English language article. (iv) Location of the study was an urban setting in SEA countries. The exclusion criteria were protocol study, conference proceedings, review articles, non-peer-reviewed articles, case studies, and animal studies. We assessed the quality of all studies included in the analysis independently using the Newcastle–Ottawa scale (NOS) according to the Cochrane Handbook for Systematic Reviews of Interventions [12, 13]. The results of the quality assessment are shown in Table 1.

### 2.3. Data Extraction

The data extracted from the eligible studies were the surname of the first author, year of publication, title and study objectives, location of research, study design, sample size, the number of positives cases, prevalence of hypertension, and the risk factors. The extracted data are shown in Table 2.

### 2.4. Data Collation and Analysis

All data were first entered in Microsoft Excel version 2019 and underwent meta-analysis using Review Manager 5 software version 5.3.5. The prevalence of hypertension for individual studies was determined by multiplying the ratio of cases to the sample size by 100. The 95% confidence interval (95% CI) was established with formula 1.96 × SQRT (*p* × (1 − *p*)/*n*), where *p* is the prevalence and *n* is the sample size. The pooled prevalence estimates (PPEs) and their 95% CI were determined using the random-effects model, based on the assumption that the true effect sizes might differ within eligible studies. Heterogeneity, which is the measure of variability between studies, was analysed using Cochran's Q-test. The percentage of variation in prevalence estimate due to heterogeneity was quantified using formula *I*^2^ = 100 × (*Q* − d*f*)/*Q*, whereby *Q* is Cochran's heterogeneity statistic and df is the degree of freedom, which is the difference between the number of studies and 1. I2 values of 0%, 25%, 50%, and 75% were considered to indicate no, low, moderate, and high heterogeneity, respectively.

## 3. Results and Discussion

### 3.1. Eligible Studies

**Figure 1:**
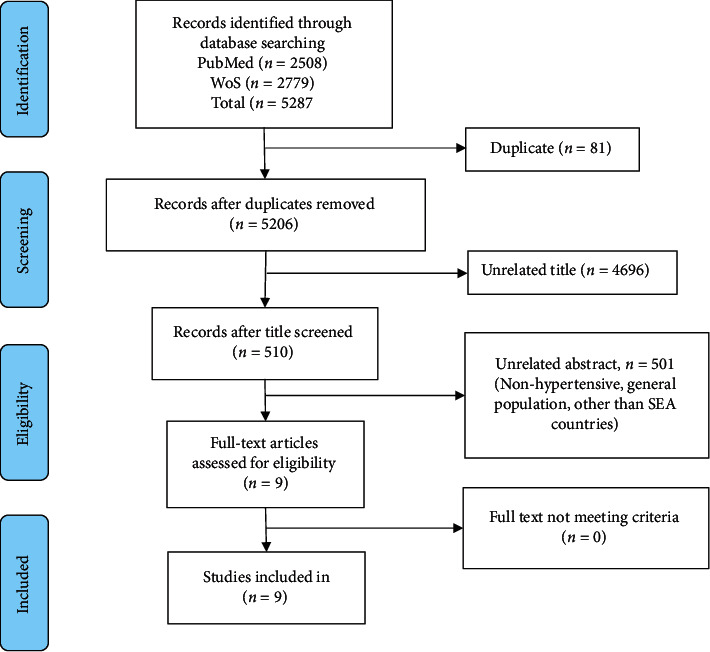
Flow diagram for the selection process of the eligibility study.

The database search generated 5287 studies, as presented in Figure 1. Eighty-one duplicate studies were removed, and a further 4696 studies were removed after the screening of titles. Five hundred and ten studies underwent abstract screening, which resulted in only nine studies being included for full-text review.

### 3.2. Characteristics of the Eligible Studies

We included nine articles in the study. Of these, two were published in 2016, two in 2017, two in 2018, and three in 2019. Eight studies were cross-sectional studies, and only one used a prospective cohort study design. All studies were conducted in the SEA region: three in Malaysia [14, 18, 19], two in Myanmar [15, 17], and one each from Singapore [20], Thailand [21], Indonesia [16], and the Philippines [22]. Based on study settings, six studies were conducted among a community [14–17, 21, 22], two were school based [18, 19] and one was in a clinic setting [22]. For the targeted population, two studies were conducted among school-going adolescents [18, 19], two were among those aged > 15 years [15, 16], and the remaining five studies were conducted among adults [14, 16, 17, 21, 22]. All the studies have their diagnostic criteria for hypertension, as shown in Table 2. A majority of the studies measured the blood pressure in the upper arms of seated patients, using a digital sphygmomanometer with a minimum of two readings.

### 3.3. Pooled Prevalence Estimates and Heterogeneity Analysis

Of the nine articles, only eight were included for meta-analysis of the PPE [14–20, 22]. One article was not included in the meta-analysis as the whole sample was hypertensive [21]. That article was used for risk factor analysis only.

**Figure 2:**
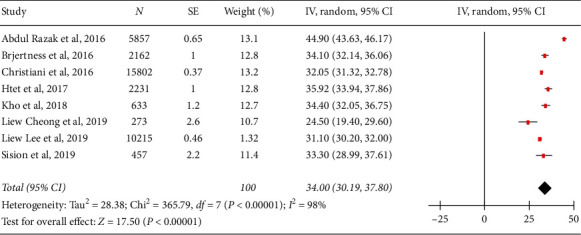
Forest plot representation of the overall studies.

**Figure 3:**
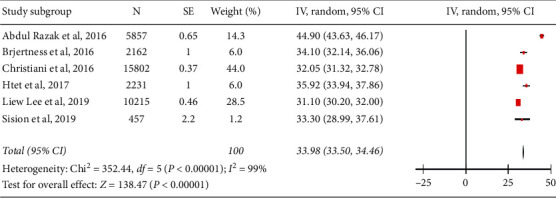
Forest plot of the studies among a community setting.

**Figure 4:**
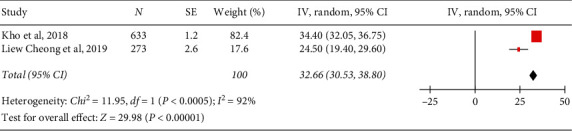
Forest plot of the studies among a school setting.

The PPEs of hypertension in the urban population are presented in Table 3. Figures 2–4 show forest plots of the overall study and based on the study setting. A total of 37,630 individuals were examined during the period under review; 12,842 had hypertension, yielding an overall PPE of 34.14% (95% CI: 30.19–37.80). The PPEs for the community-based and school-based study settings were 33.98% (95% CI: 33.50–34.46) and 32.66% (95% CI: 30.53–34.80), respectively. A high degree of heterogeneity was observed within the studies and subgroups (Table 2), indicating that the data were less conclusive. The prevalence of hypertension among adolescents in school-based settings was slightly lower than that in adults in community-based settings. This was very much expected because adolescents have less risk of developing hypertension due to their young age. However, the 32.66% PPE among urban adolescents is alarming, as it indicates that almost one-third of them have hypertension.

### 3.4. Associated Factors of Hypertension among the Urban Population

Of the nine articles, seven were synthesized for risk or associated factors of hypertension in the urban population, as demonstrated in Table 4. The remaining two articles [14, 18] did not mention the risks or associated factors specific to the urban population. Two articles [15, 20] mentioned that being male was a risk for developing hypertension in urbanites. With regards to age, Cristiani et al. [16] mentioned that men aged < 45 years and women aged > 45 years have higher risk for hypertension. Based on the location of their studies, Cristiani et al. [16] and Liew et al. [20] showed that certain ethnicities were associated with hypertension. The study in Singapore showed that Malays had a higher risk of hypertension, while the study in Sarawak showed that being Chinese and Iban were risk factors for hypertension. That study also confirmed that low education and socioeconomic levels were associated with hypertension among the urban population. The other associated factors were high BMI and increased waist circumference [16], dyslipidaemia [16, 22], and smoking [22].

## 4. Discussion

We found that the PPE of hypertension among adults in Asia is 33.98% (95% CI: 33.50–34.46), and that it is slightly higher than the global prevalence of hypertension (31.1%) [23]. In another global study, the prevalence of hypertension was from as low as 3.4% in India and as high as 72.5% in Poland [24]. However, that study did not stratify the population into urban and rural. Another systematic review specifically of the urban population in middle- and low-income countries reported that the PPE of hypertension was 32.7% (95% CI: 30.4–35.0) [25], which was similar to our results. The urban population in Latin America and the Caribbean had the highest PPE of hypertension, i.e. 51.2% (95% CI: 30.1–72.0), followed by that of East Asia and the Pacific, i.e. 51.2% (95% CI: 32.8–69.5), and South Asia, i.e. 35.9% (95% CI: 19.1–54.7).

In Asia, the prevalence of hypertension in urban adult populations is 15–35% [26]. A more recent study reported a lower prevalence of hypertension of 31.2% in urban South Asia [27]. For SEA specifically, a comprehensive review reported an adult hypertension prevalence of 35% [28], slightly higher than that reported in the present study. One plausible explanation is that these studies included different countries in the SEA region in comparison to our study, and there was no rural-urban stratification.

The PPE of hypertension among urban adolescents reported in the present study was relatively high, being about 32.45% (95% CI: 30.31–34.58). This result is only slightly lower than that for the adult population. A meta-analysis performed in Africa reported a relatively low PPE among adolescents: 5.5% (95% CI: 4.2–6.9). Countries such as Brazil and Iran have reported a relatively similar PPE of hypertension among adolescents (8.12% (95% CI: 6.24–10.52) and 8.9 (95% CI: 7.5–10.3), respectively) [29, 30]. A study in Pakistan reported a higher adolescent urban PPE of hypertension in comparison to the studies mentioned above: 26.61% (21.80%, 31.42%) [31].

The risk factors for hypertension can be divided into sociodemographic and modifiable risk factors. The sociodemographic risk factors described in the present review are sex, ethnicity, education, and socioeconomic status. In our review, two articles [15, 21] mention that being male is a risk factor of developing hypertension. Similarly, a systematic review in Asia showed that a study in Southern China and Cameroon noted a significantly higher prevalence of hypertension among men in comparison to women [32–34]. However, a meta-analysis performed globally in the middle- and lower-income countries showed that there was no significant sex difference in hypertension prevalence (31.9% vs. 30.8%, *P* = 0.6) [26]. Another meta-analysis performed in South Asia also noted no difference between the prevalence of hypertension between men and women (men: 31% (standard deviation, SD = 7.15); women (*N* = 10), 31%). In contrast, in the United States, the prevalence of hypertension was higher in women (30.1%) in comparison to men (27.1%) [28].

In the present review, the study locations showed that certain ethnicities were associated with hypertension in the urban population [16, 21]. According to the US Centres for Disease Control and Prevention (CDC), black people have a higher prevalence of developing high blood pressure than white people, Hispanics, Asians, Pacific Islanders, Native Americans, or Alaska Natives. In comparison to white people, black people also develop hypertension earlier in life [35]. A study performed in an Asian country showed a higher prevalence of hypertension among Malays in comparison with the Chinese and Indians [36].

In the present study, low education is associated with hypertension among the urban population [16, 21]. A few studies performed in China also reported similar findings, where those with lower education in the urban population had a higher risk of hypertension [37, 38]. Similarly, in a meta-analysis performed globally, those without formal education had significantly higher prevalence of hypertension in comparison to those who had formal education [26]. According to the World Health Organization (WHO), social determinants of health such as education have an impact on behavioural risk factors [39]. Higher education may aid health literacy of the importance of a healthy lifestyle, which includes a healthy diet, physical activity, and regular checkups, which lower the risk of developing hypertension [37, 40].

In our review, low socioeconomic level was associated with hypertension among the urban population [16, 21]. People with low socioeconomic status might be unemployed, and unemployment has an impact on stress levels, leading to high blood pressure [41]. Moreover, people with low socioeconomic status might have jobs with fewer health benefit packages, which results in overall poorer health status [37]. On the other hand, one study stated that the PPE for hypertension was also the highest across upper middle-income countries (37.8%, 95% CI: 35.0–40.6) and the lowest across low-income countries (23.1%, 95% CI: 20.1–26.2) [26]. Similarly, a few studies in China noted that those with higher job positions had higher risk of hypertension [38, 40]. People with higher job positions have higher stress levels, which lead to hypertension. This group of people might also have jobs that involve sitting and using computers, which lead to reduced physical activity and eventually lead to hypertension.

The other associated factors of hypertension reported in the present study are high BMI and increased waist circumference [19], dyslipidaemia [16, 22], and smoking [22]. The local public health systems in every country should implement interventions of these modifiable factors.

Our findings show that high BMI plays a crucial role as a determinant of hypertension [20]. In a meta-analysis conducted globally, people who were obese had a higher risk of developing hypertension in comparison to those with normal weight [26]. Similarly, a meta-analysis performed in South Asia showed that obesity was statistically significant for the development of hypertension in comparison to normal weight [27]. In fact, observational studies performed in urban settings in Kenya (adjusted odds ratio, AOR: 1.8; 95% CI: 1.1–3.1), China (AOR: 1.91; 95% CI: 1.76–2.07), and Cameroon (AOR: 1.59; 95% CI: 1.45–1.73) agree that being obese or overweight is twice more likely to be associated with hypertension compared to being nonobese [34, 40–42].

Many studies have reported that excessive body weight is a major cause of hypertension, accounting for 65–75% of the risk for primary (essential) hypertension. The pathophysiology lies in the increased renal tubular sodium reabsorption that impairs pressure natriuresis and plays an important role in initiating obesity hypertension. There are three factors of abnormal kidney function and increased blood pressure during the development of obesity hypertension: (1) physical compression of the kidneys by fat around the kidneys. (2) Activation of the renin-angiotensin-aldosterone system. (3) Increased sympathetic nervous system activity. Controlling obesity-associated hypertension becomes more challenging with prolonged obesity and the development of target organ injury, especially renal damage. Subsequently, multiple antihypertensive drugs and the treatment of other modifiable risk factors are often required [43].

Interestingly, instead of being a disease burden, the presence of hypertension and some degrees of elevated BMI and serum cholesterol are associated with lower risk of death among the geriatric population. This phenomenon is termed ‘reverse epidemiology' or ‘risk factor paradox'. It is also observed in a variety of chronic disease states such as end-stage kidney disease that requires dialysis, chronic heart failure, rheumatoid arthritis, and AIDS. Several possible causes have been hypothesised to explain this reverse epidemiology, such as competing short-term and long-term killers, improved hemodynamic stability in the obese, adipokine protection against tumour necrosis factor *α*, lipoprotein protection against endotoxins, and lipophilic toxin sequestration by adipose tissue [44].

Two articles included in our study reported on high waist circumference as a risk factor for hypertension [20]. A meta-analysis performed in South Asia found that high waist circumference was statistically significant for the development of hypertension [27]. Many urban hypertensive studies performed globally have also indicated that high waist circumference carries 2–3 times higher risk of being associated with hypertension as compared to normal waist circumference. Examples of such findings are three studies conducted in China (AOR: 1.66; 95% CI: 1.54–1.80), Hong Kong (AOR: 2.38, 95% CI: 1.13–4.99), and the United States (AOR: 2.79; 95% CI: 1.44–5.41) [42, 45, 46]. Furthermore, many studies have also revealed that the combination of high waist circumference and high BMI is superior to individual indices for predicting hypertension [33, 47].

The next modifiable factor we found is dyslipidaemia, as reported in three articles included our study [16, 22]. Various epidemiological studies have shown that the prevalence of the coexistence of hypertension and dyslipidaemia is 15–31%. The coexistence of the two risk factors has more than an additive adverse impact on the vascular endothelium, resulting in enhanced atherosclerosis. Although many studies have shown that dyslipidaemia does not carry very high odds of developing hypertension, it is nevertheless very much reported to have a significant association with hypertension and other cardiovascular diseases [48, 49]. For example, a study conducted in Zambia showed that high serum cholesterol presented a slight risk of hypertension as compared to normal serum cholesterol levels (AOR: 1.30; 95% CI: 1.14, 1.48) [50]. On the other hand, hypertension also serves as a predictor for dyslipidaemia. One such example is shown in an Iranian study, where hypertensive people are twice as likely to have dyslipidaemia (AOR: 1.62; 95% CI: 1.42–1.83) as compared to nonhypertensive people [51].

Finally, we found only one article that reported on smoking as an associated factor of hypertension [22]. In a meta-analysis performed in South Asia, the pooled OR showed that smokers had a likelihood of having hypertension [27]. Many studies have shown that the association of smoking with the development of hypertension is diverse in different urban settings. For example, a study conducted in India revealed that smoking was six times more highly associated with hypertension (AOR: 6.4; 95% CI: 1.9–20.9), while tobacco chewing was seven times more highly associated with hypertension (AOR: 6.8; 95% CI: 1.9–20.9) as compared to no consumption of any tobacco products [52]. A lower association was evident in studies conducted in Nepal (AOR: 1.957; 95% CI: 1.219–3.141) and China (AOR: 1.28; 95% CI: 1.17–1.39), where smokers carried a slight risk of developing hypertension as compared to nonsmokers or those who had already quit [42, 53]. Nonetheless, a study in Zambia reported that there is no significant difference in either being a smoker or nonsmoker (AOR: 0.97; 95% CI: 0.81–1.17) [50].

Since meta-analysis forms the highest level of evidence, it is essential that the quality assessment is performed precisely by standard tools [36]. The quality assessment was performed in this study to ensure the overall strength of evidence and methodological quality of the research design with respect to the research question [37]. The results of meta-analysis are dependent on the evaluation of quality of studies [36]. In this systematic review, the quality assessment was performed via the Newcastle–Ottawa scale (NOS) which has several advantages, namely, being relatively fast, being a validated tool, and the presence of scoring which can be used as a moderator in meta-regression.

## 5. Strengths and Limitation

To our knowledge, this is the first systematic review and meta-analysis of the SEA urban population to determine the prevalence of hypertension. Many different SEA countries were included in this study, namely, Malaysia, Singapore, Thailand, Indonesia, and the Philippines. Besides that, the study involved both adult and adolescent populations, allowing for further subgroup analysis, despite there being only two articles on hypertension and adolescents. We only determined the urban prevalence of hypertension and made no rural-urban comparison, which is a study limitation. Moreover, that the meta-analysis was performed on the urban population also limits generalisation of the findings, as SEA is composed of many large cities with a large proportion of rural areas. Generalisation also cannot be made, as our study involved only five of the total 10 SEA countries. Finally, our search criteria were limited to only two research databases and to articles written in English only.

## 6. Conclusions

Our findings reveal an essential healthcare issue among urban SEA adults and adolescents. Here, we found that about 1 in 3 adults and adolescents in SEA are hypertensive. Clearly, the rate of hypertension, especially among adolescents, is developing at an alarming rate. Our findings encourage urgent primary and secondary prevention activities and may have implications for policy and intervention development among policymakers. Health education and proper screening need to be carried out not only for the general population but also for secondary school adolescents. Urbanisation is unavoidable and should not be blamed for the prevalence of hypertension. Therefore, there should be a multisectoral and intersectoral approach and collaboration for improving the overall health outcomes of all populations in all SEA countries.

## Figures and Tables

**Table 1:** Quality assessment of selected articles.

Authors (years)	Selection (maximum 5 stars)				Comparability (maximum 2 stars)	Outcome (maximum 3 stars)		Total stars
	Representativeness of the sample	Sample size	Nonrespondent	Ascertainment of the exposure (risk factor)	The subjects in different outcome groups are comparable, based on the study design or analysis. Confounding factors are controlled	Assessment of the outcome	Statistical test	
Abdul-Razak et al. (2016) [14]	1	1	0	2	1	2	1	8
Bjertness et al. (2016) [15]	1	1	0	2	1	2	1	8
Christiani et al. (2016) [16]	1	1	0	1	1	2	1	7
Htet et al. (2017) [17]	1	1	1	1	1	2	1	8
Kho et al. (2018) [18]	1	1	1	1	1	2	1	8
Liew et al. (2019) [19]	0	1	1	2	1	1	1	7
Liew et al. (2019) [20]	1	1	1	2	1	1	1	8
Visanuyothin et al. (2018) [21]	1	1	1	2	1	2	1	9
Sison et al. (2019) [22]	1	1	1	2	0	2	1	8

**Table 2:** Study characteristic, the prevalence of hypertension.

No.	Author (year)	Type of study	Country/state/region of study	Study setting	Sample population/age	Sample size	Subgroup of population (if any)	No. of positive cases of HPT	Overall prevalence of HPT, % (with 96% CI)	Subgroup prevalence, % (with 96% CI)	Diagnostic criteria of HPT
(1)	Abdul-Razak et al. (2016) [14]	Prospective cohort study	Selangor, Negeri Sembilan, Pahang, Kelantan, Sarawak and Sabah, and the Federal territory of Kuala Lumpur (Malaysia)	Community	Adults aged ≥30 years	Overall *N* = 11267, urban *N* = 5857 (51.9%), and rural *N* = 5410 (48.1%)	There was an overall subgroup (age, sex, race, education level, smoking status, and obesity); however, it was unable to specify the urban subgroup	Overall 5409, urban: 2630, rural	Overall 47.9 (47.0–49.0), urban: 44.9 (43.6–46.2), rural 51.2 (49.8–52.4)	NA	(1) Systolic BP ≥ 140 mmHg and/or diastolic BP ≥ 90 mmHg (measured twice using an automatic digital blood pressure monitor); (2) the participants reported a history of hypertension; or (3) the participants reported taking antihypertensive medications in the past two weeks
(2)	Bjertness et al. (2016) [15]	Cross sectional	Myanmar	Community	Adults 15–64 years	Overall *N* = 7319, urban *N* = 2162 (29.5%), rural *N* = 5157 (70.5%)	(Urban only) male *N* = 851, Female *N* = 1311. Another subgroup was overall and not specific to the urban population	Overall 2171, urban 737, rural 1434	Overall 29.9 (28.8–31.0), urban 34.1 (32.1–36.1), rural 27.8 (26.6–29.0)	(Urban only) male 30.1 (27.0–33.2) Female 29.8 (27.3–32.3)	(1) Systolic BP ≥ 140 mmHg and/or average diastolic BP ≥ 90 mmHg (measured three times using an automatic digital blood pressure monitor); (2) or participants reported taking antihypertensive medications
(3)	Christiani et al. (2016) [16]	Cross sectional	Indonesia	Community in urban	Adults > 15 years	15,802 urban population (7543 males and 8259 females)	Male-female, by age	5064, male-2429; female 2635	32.1 (31.32–32.77)	Male: 32.2 (31.47–32.93); Female: 31.9 (31.53–32.27)	Systolic BP > 140 mmHg or mean diastolic BP was >90 mmHg on 3 blood pressure measurements (did not mention the tool used for BP measurement)
(4)	Htet et al. (2017) [15]	Cross-sectional	Yangon, Myanmar	Community	Adults (25–74 years old)	Total subjects in urban and rural: 2004 (*N* = 4448), 2014 (*N* = 1486). For the year 2014, the sample size for urban was 2231	Urban rural, male-female. For the year 2014, the male urban was 745 and the female urban was 1486	2004: male-556, female-680; 2014: male-288, female-256. In 2014, 801 out of 2231 urban population had HPT	2004: 27.6 (24.3–31.1) 2014: 34.5 (31.5–37.6). In 2014, the prevalence of HPT in urban was 35.9	HPT for male in 2004: 27.9 (23.9–32.4), HPT of male in 2014: 38.7 (33.9–43.7), HPT for female in 2004:27.7 (24.4–31.3) HPT for female in 2004 34.5 (28.9–40.6). In 2014, urban men and women had 38.7 and 34.5 of HPT, respectively	(1) Systolic BP ≥ 140 mmHg and/or average diastolic BP ≥ 90 mmHg (measured three times using an automatic digital blood pressure monitor); (2) participants reported taking antihypertensive medications in the past two weeks
(5)	Kho et al. (2018) [16]	Cross sectional	Sarawak, Malaysia	School based	Adolescents (12–17 years old)	*N* = 2461 (total), *n* = 633 (urban).		*n* = 218 (urban), *n* = 523 (rural).	30.1 (29.84–30.64) (total)	34.4 (34.14–34.66) (urban).	Hypertension was defined as an average systolic BP and/or diastolic BP above or equal to the 95^th^ percentile for sex, age, and height (measured three times using an automatic digital blood pressure monitor)
(6)	Liew et al. (2019) [17]	Cross sectional	Johor Bahru, Malaysia	School based	Adolescents (mean 14.6 years of age)	*N* = 273	NA	*n* = 67	24.5 (19.40–29.60)	NA	Hypertension was defined as a systolic BP or diastolic BP above the 9th percentile (measured three times using an automatic digital blood pressure monitor)
(7)	Liew et al. (2019) [18]	Cross sectional	Singapore	Community	Adults ≥21 years	*N* = 10215	NA	3177	31.1 (30.20–32.00)	NA	1) Systolic BP ≥ 140 mmHg and/or diastolic BP ≥ 90 mmHg (measured twice using an automatic digital blood pressure monitor); (2) the participants reported a history of hypertension
(8)	Visanuyothin et al. (2018) [19]	Cross sectional	Thailand	Community in urban	Adults (median 63 years old)	125	All hypertensive patients are divided into a controlled and uncontrolled group	73 (well controlled)	58.4 (58.31–58.47)	Uncontrolled hypertension: 41.6 (41.51–41.67)	BP ≥ 140/90 mmHg in last three visits, measured using an automatic digital blood pressure monitor
(9)	Sison et al. (2019) [20]	Cross sectional	The Philippines	Community healthcare workers	Adults (mean 49.3 years of age)	457	Urban rural, male-female	148	32.4 (32.34–32.44)	Urban: 33.3 (33.24–33.36)	(1) Systolic BP ≥ 140 mmHg and/or diastolic BP ≥ 90 mmHg (measured three times using an automatic digital blood pressure monitor); (2) participants reported taking antihypertensive medications

**Table 3:** Pooled prevalence estimates for hypertension among the urban population in SEA countries.

Variables	No. of studies	Pooled prevalence estimates			95% CI	Heterogeneity	
		Sample size	HPT case	Prevalence (%)		*I* ^2^ (%)	*Q* − *P*
Overall	8	37630	12842	34.14	30.19–37.80	98	<0.001
Study setting							
Community based	6	36724	12557	33.98	33.50–34.46	99	<0.001
School based	2	906	285	32.66	30.53–34.80	92	<0.001

**Table 4:** Associated risk factors for hypertension identified in each study.

No.	Author (year)	Risk factors										Notes
		Male gender	Older age	Ethnicity	Lower education level	Smoking	Alcohol	High BMI	High WC	High cholesterol	DM	
(1)	Bjertness et al. (2016) [15]	+	+	N	N	N	+	+	+	N	N	The only specific risk factor for urban was being male. Others were overall risk factors
(2)	Christiani et al. (2016) [16]	−	+	N	+	+	N	+	N	+	N	Women have a higher risk of raised blood pressure at an age of ≥45 years. Men have a higher risk of raised blood pressure at an age of 15 to ≤45 years
(3)	Htet et al. (2017) [17]	+	N	N	N	N	+	+	N	N	+	The only specific risk factor for urban was being male. Others were overall risk factors
(4)	Liew et al. (2019) [19]	N	N	N	N	N	+	+	N	N	N	
(5)	Liew et al. (2019) [20]	+	N	+ (Malay)	+	N	N	N	N	N	N	Additional risk factor: homemaker
(6)	Visanuyothin et al. (2018) [21]	N	N	N	N	+	N	N	N	+	N	Smoking history and having hyperlipidemia
(7)	Sison et al. (2019) [22]	+	+	N	N	N	+	+	N	N	N	The only specific risk factor for urban was being male. Others were overall risk factors

N = not measured, + = positively associated with hypertension, − = negatively associated with hypertension.

## Data Availability

The authors declare that the data supporting the findings of this study are available within the article.

## References

[1] World Health Organization (2019). Hypertension. https://www.who.int/news-room/fact-sheets/detail/hypertension.

[2] Kingue S., Ngoe C. N., Menanga A. P. (2015). Prevalence and risk factors of hypertension in urban areas of Cameroon: a nationwide population-based cross-sectional study. *The Journal of Clinical Hypertension*.

[3] Lamelas P., Diaz R., Orlandini A. (2019). Prevalence, awareness, treatment and control of hypertension in rural and urban communities in Latin American countries. *Journal of Hypertension*.

[4] Flynn J. T., Kaelber D. C., Baker-Smith C. M. (2017). Subcommittee on screening and management of high blood pressure In children. Clinical practice guideline for screening and management of high blood pressure in children and adolescents. *Pediatrics*.

[5] DiPietro A., Kees-Folts D., DesHarnais S. (2009). Primary hypertension at a single center: treatment, time to control, and extended follow-up. *Pediatric Nephrology*.

[6] Univeristy NI (2015). Center for Southeast Asian studies. https://www.niu.edu/cseas/resources/countries.shtml.

[7] World Health Organization (2018). Noncommunicable diseases country profiles. https://www.who.int/nmh/publications/ncd-profiles-2018/en/.

[8] Kaddumukasa M., Kayima J., Nakibuuka J. (2017). Modifiable lifestyle risk factors for stroke among a high risk hypertensive population in Greater Kampala, Uganda; a cross-sectional study. *BMC Research Notes*.

[9] Xing L., Jing L., Tian Y. (2019). Urban–rural disparities in status of hypertension in northeast China: a population-based study, 2017–2019. *Clinical Epidemiology*.

[10] Singh S., Shankar R., Singh G. P. (2017). Prevalence and associated risk factors of hypertension: a cross-sectional study in urban Varanasi. *International Journal of Hypertension*.

[11] Moher D., Liberati A., Tetzlaff J., Altman D. G., Group P. (2009). Preferred reporting items for systematic reviews and meta-analyses: the PRISMA statement. *PLoS Medicine*.

[12] Wells G., Shea B., O’connell D. (2014). *The Newcastle-Ottawa Scale (NOS) for Assessing the Quality of Nonrandomised Studies in Meta-Analyses*.

[13] Higgins J. P., Green S. (2011). *Cochrane Handbook for Systematic Reviews of Interventions*.

[14] Abdul-Razak S., Daher A. M., Ramli A. S. (2016). Prevalence, awareness, treatment, control and socio demographic determinants of hypertension in Malaysian adults. *BMC Public Health*.

[15] Bjertness M. B., Htet A. S., Meyer H. E. (2016). Prevalence and determinants of hypertension in Myanmar—a nationwide cross-sectional study. *BMC Public Health*.

[16] Christiani Y., Byles J. E., Tavener M., Dugdale P. (2016). Gender inequalities in noncommunicable disease risk factors among Indonesian urban population. *Asia-Pacific Journal of Public Health*.

[17] Htet A. S., Bjertness M. B., Oo W. M. (2017). Changes in prevalence, awareness, treatment and control of hypertension from 2004 to 2014 among 25–74-year-old citizens in the Yangon region, Myanmar. *BMC Public Health*.

[18] Kho W. F. G., Cheah W. L., Hazmi H. (2018). Elevated blood pressure and its predictors among secondary school students in Sarawak: a cross-sectional study. *Central European Journal of Public Health*.

[19] Liew J. K., Cheong X. P., Law L. (2019). Prevalence and factors associated with hypertension among adolescents in Malaysia. *IMJM*.

[20] Liew S. J., Lee J. T., Tan C. S., Koh C. H. G., Van Dam R., Muller-Riemenschneider F. (2019). Sociodemographic factors in relation to hypertension prevalence, awareness, treatment and control in a multi-ethnic Asian population: a cross-sectional study. *BMJ Open*.

[21] Visanuyothin S., Plianbangchang S., Somrongthong R. (2018). Appearance and potential predictors of poorly controlled hypertension at the primary care level in an urban community. *Journal of Multidisciplinary Healthcare*.

[22] Sison O., Castillo-Carandang N., Ladia M. A. (2019). Prevalence of metabolic syndrome and cardiovascular risk factors among community health workers in selected villages in the Philippines. *Journal of the ASEAN Federation of Endocrine Societies*.

[23] Mills K. T., Bundy J. D., Kelly T. N. (2016). Global disparities of hypertension prevalence and control: a systematic analysis of population-based studies from 90 countries. *Circulation*.

[24] Kearney P. M., Whelton M., Reynolds K., Whelton P. K., He J. (2004). Worldwide prevalence of hypertension: a systematic review. *Journal of Hypertension*.

[25] Sarki A. M., Nduka C. U., Stranges S., Kandala N.-B., Uthman O. A. (2015). Prevalence of hypertension in low-and middle-income countries: a systematic review and meta-analysis. *Medicine*.

[26] Singh R., Suh I., Singh V. (2000). Hypertension and stroke in Asia: prevalence, control and strategies in developing countries for prevention. *Journal of Human Hypertension*.

[27] Neupane D., McLachlan C. S., Sharma R. (2014). Prevalence of hypertension in member countries of South Asian Association for Regional Cooperation (SAARC): systematic review and meta-analysis. *Medicine*.

[28] Krishnan A., Garg R., Kahandaliyanage A. (2013). Hypertension in the South-east Asia region: an overview. *Regional Health Forum*.

[29] Magliano E. S., Guedes L. G., Coutinho E. S. F., Bloch K. V. (2013). Prevalence of arterial hypertension among Brazilian adolescents: systematic review and meta-analysis. *BMC Public Health*.

[30] Akbari M., Moosazadeh M., Ghahramani S. (2017). High prevalence of hypertension among Iranian children and adolescents: a systematic review and meta-analysis. *Journal of Hypertension*.

[31] Shah N., Shah Q., Shah A. J. (2018). The burden and high prevalence of hypertension in Pakistani adolescents: a meta-analysis of the published studies. *Archives of Public Health*.

[32] Aryal N., Wasti S. P. (2016). The prevalence of metabolic syndrome in South Asia: a systematic review. *International Journal of Diabetes in Developing Countries*.

[33] Hu L., Huang X., You C. (2017). Prevalence and risk factors of prehypertension and hypertension in Southern China. *PLoS One*.

[34] Kingue S., Ngoe C. N., Menanga A. P. (2015). Prevalence and risk factors of hypertension in urban areas of Cameroon: a nationwide population‐based cross‐sectional study. *Journal of Clinical Hypertension (Greenwich)*.

[35] Centers of Disease Control and Prevention (2020). High blood presure: know your risk of high blood pressure 2020. https://www.cdc.gov/bloodpressure/risk_factors.htm.

[36] Sabanayagam C., Teo B. W., Tai E. S., Jafar T. H., Wong T. Y. (2013). Ethnic differences in the association between blood pressure components and chronic kidney disease in middle aged and older Asian adults. *BMC Nephrology*.

[37] Wang Y., Chen J., Wang K., Edwards C. (2006). Education as an important risk factor for the prevalence of hypertension and elevated blood pressure in Chinese men and women. *Journal of Human Hypertension*.

[38] Meng X.-J., Dong G.-H., Wang D. (2011). Prevalence, awareness, treatment, control, and risk factors associated with hypertension in urban adults from 33 communities of China: the CHPSNE study. *Journal of Hypertension*.

[39] World Health Organization (2013). A global brief on hypertension. silent killer, global public health crisis: world health day. https://apps.who.int/iris/handle/10665/79059.

[40] Shen Y., Chang C., Zhang J., Jiang Y., Ni B., Wang Y. (2017). Prevalence and risk factors associated with hypertension and prehypertension in a working population at high altitude in China: a cross-sectional study. *Environmental Health and Preventive Medicine*.

[41] Olack B., Wabwire-Mangen F., Smeeth L., Montgomery J. M., Kiwanuka N., Breiman R. F. (2015). Risk factors of hypertension among adults aged 35–64 years living in an urban slum Nairobi, Kenya. *BMC Public Health*.

[42] Tian S., Dong G.-H., Wang D. (2011). Factors associated with prevalence, awareness, treatment and control of hypertension in urban adults from 33 communities in China: the CHPSNE Study. *Hypertension Research*.

[43] Hall J. E., do Carmo J. M., da Silva A. A., Wang Z., Hall M. E. (2015). Obesity-induced hypertension: interaction of neurohumoral and renal mechanisms. *Circulation Research*.

[44] Ahmadi S.-F., Streja E., Zahmatkesh G. (2015). Reverse epidemiology of traditional cardiovascular risk factors in the geriatric population. *Journal of the American Medical Directors Association*.

[45] Leung L. C., Sung R. Y., So H.-K. (2011). Prevalence and risk factors for hypertension in Hong Kong Chinese adolescents: waist circumference predicts hypertension, exercise decreases risk. *Archives of Disease in Childhood*.

[46] Warren T. Y., Wilcox S., Dowda M., Baruth M. (2012). Peer reviewed: independent association of waist circumference with hypertension and diabetes in African American women, South Carolina, 2007–2009. *Preventing Chronic Disease*.

[47] Kalani Z., Rafiei M., Salimi T. (2015). Comparison of obesity indexes BMI, WHR and WC in association with hypertension: results from a blood pressure status survey in Iran. *Journal of Cardiovascular Disease Research*.

[48] Dalal J. J., Padmanabhan T., Jain P., Patil S., Vasnawala H., Gulati A. (2012). LIPITENSION: interplay between dyslipidemia and hypertension. *Indian Journal of Endocrinology and Metabolism*.

[49] Nelson R. H. (2013). Hyperlipidemia as a risk factor for cardiovascular disease. *Primary Care*.

[50] Goma F. M., Nzala S. H., Babaniyi O. (2011). Prevalence of hypertension and its correlates in Lusaka urban district of Zambia: a population based survey. *International Archives of Medicine*.

[51] Ebrahimi H., Emamian M. H., Hashemi H., Fotouhi A. (2016). Dyslipidemia and its risk factors among urban middle-aged Iranians: a population-based study. *Diabetes & Metabolic Syndrome: Clinical Research & Reviews*.

[52] Bhadoria A. S., Kasar P. K., Toppo N. A., Bhadoria P., Pradhan S., Kabirpanthi V. (2014). Prevalence of hypertension and associated cardiovascular risk factors in Central India. *Journal of Family and Community Medicine*.

[53] Dhungana R. R., Pandey A. R., Bista B., Joshi S., Devkota S. (2016). Prevalence and associated factors of hypertension: a community-based cross-sectional study in municipalities of Kathmandu, Nepal. *International Journal of Hypertension*.

